# Efficacy and Safety of an Anti-nerve Growth Factor Antibody (Frunevetmab) for the Treatment of Degenerative Joint Disease-Associated Chronic Pain in Cats: A Multisite Pilot Field Study

**DOI:** 10.3389/fvets.2021.610028

**Published:** 2021-05-28

**Authors:** Margaret E. Gruen, Jamie A. E. Myers, B. Duncan X. Lascelles

**Affiliations:** ^1^Translational Research in Pain Program, Department of Clinical Sciences, College of Veterinary Medicine, North Carolina State University, Raleigh, NC, United States; ^2^Behavioral Medicine, Department of Clinical Sciences, College of Veterinary Medicine, North Carolina State University, Raleigh, NC, United States; ^3^Comparative Pain Research and Education Center, North Carolina State University, Raleigh, NC, United States; ^4^Veterinary Medicine Research and Development, Zoetis, Inc., Kalamazoo, MI, United States; ^5^Thurston Arthritis Center, University of North Carolina (UNC) School of Medicine, Chapel Hill, NC, United States; ^6^Department of Anesthesiology, Center for Translational Pain Research, Duke University, Durham, NC, United States

**Keywords:** feline, osteoarthritis, DJD, monoclonal antibody, mobility, pain

## Abstract

**Background:** Pain management for cats with degenerative joint disease (DJD) remains a critical unmet need. Recent work has shown promise for a feline-specific anti-nerve growth factor monoclonal antibody (frunevetmab) to deliver safe and effective pain management. Our objectives were to evaluate the efficacy and safety of frunevetmab administered twice using two administration routes (subcutaneous and intravenous) compared to placebo.

**Methods:** This was a randomized placebo-controlled, double-masked study. After a week-long pain and activity baseline, 126 cats were randomized to receive injections of frunevetmab (IV then SC; *n* = 42 or SC then SC; *n* = 43) or placebo (IV then SC; *n* = 41) on Days 0 and 28. Owners completed questionnaires on Days 14, 28, 42, and 56. Accelerometry data were collected continuously throughout.

**Results:** Owner questionnaire results showed significant improvement in frunevetmab-treated cats [compared to placebo; (*p* < 0.05)] at Days 42 and 56; no difference was found between routes of administration for frunevetmab. All groups had decreased objectively measured weekly activity from baseline; frunevetmab-treated cats had a mean decrease of 0.9%, while placebo-treated cats had a mean decrease of 9.3%. Treatments were generally well-tolerated. The majority of adverse events included dermatitis/alopecia related to activity-monitor collars; these occurred in a higher percentage of frunevetmab, compared to placebo, treated cats.

**Conclusions and Clinical Relevance:** Treatment with frunevetmab provided improvements in owner ratings of mobility over treatment with placebo; these results were supported by objectively measured accelerometry. Frunevetmab has the potential to address a critical gap in the treatment of chronic pain in cats.

## Introduction

Degenerative joint diseases, including osteoarthritis (OA), are associated with chronic pain and are highly prevalent in cats. Prevalence studies have shown that 64–90% of cats have radiographic evidence of degenerative joint disease (DJD) and ~45% of these cats will have associated pain and clinical signs related to mobility impairment (1, 2). Over the past decade, several studies have provided evidence for the efficacy of various treatments for the pain associated with DJD in cats (3–7). These include meloxicam (3, 6), gabapentin (4), amantadine (8), and tramadol (5, 6, 9); safety has been assessed for robenacoxib (10) and grapiprant (11). However, these are typically studies with a relatively small number of cats or are located in University settings that may not be representative of most veterinary practices. In addition, most of the treatments studied have been given via the oral route, which can be problematic for many cat owners. A high percentage of cats with DJD have comorbid renal disease (12). While evidence supports the safety of non-steroidal anti-inflammatory treatments in euvolemic cats with International Renal Interest Society (IRIS) stage I–II chronic renal disease (13, 14), concerns remain about the long-term use of such medications in these cats.

There remains a critical need for treatment options for cats with DJD and associated pain. Novel therapeutic targets have been suggested, including nerve-growth factor (NGF). NGF was originally discovered as a factor in the development and maintenance of sensory and sympathetic neurons in the developing nervous system (15). However, in adults, the main role of NGF in the periphery is pro-nociceptive (15). Preclinical and clinical researches over the past several decades have clearly demonstrated the important role of NGF in rodent models and human chronic pain states, including DJD pain (15, 16). NGF is elevated in response to injury, disease, and noxious stimuli; NGF mediates its activity primarily via binding to tropomyosin receptor kinase A (TrkA), resulting in both increased sensitivity of the sensory neuron and changes in the phenotype of the neuron. Phenotypic changes include increased expression of pro-nociceptive neurotransmitters such as substance P and calcitonin gene-related peptide, which are released from the peripheral terminals and contribute to neurogenic inflammation. Released NGF also binds to TrkA located on inflammatory cells, eliciting further release of inflammatory mediators, such as histamine, serotonin, and NGF itself. Thus, NGF not only can trigger peripheral sensitization but also contributes to both neurogenic and cell-mediated inflammation (15, 17).

Anti-NGF monoclonal antibodies (mAbs) are in development as treatments for several pain conditions in multiple species. In studies performed thus far, dose-dependent efficacy was demonstrated in human patients with moderate to severe pain associated with symptomatic knee or hip OA (18–20). Efficacy appeared greater than that observed with non-steroidal anti-inflammatory drugs (NSAIDs) or opiates (21). The use of anti-NGF mAbs in companion animals has recently been reviewed (22). In dogs, a fully caninized anti-NGF mAb (ranevetmab) therapy was developed (23) and assessed for efficacy in the treatment of chronic osteoarthritic pain (24, 25). These studies in dogs showed improvement in owner-rated pain scores (24) and improvement in activity, owner-rated quality of life, and owner-rated mobility (25). Recently, the European Commission authorized a different anti-NGF mAb (bedinvetmab) for the alleviation of osteoarthritis pain in dogs^1^ In cats, a felinized anti-NGF mAb (frunevetmab) was developed and assessed for preliminary safety and efficacy in a feline model of chronic pain (26). Subsequently, a small, single-site, university-based proof-of-concept study demonstrated efficacy for this anti-NGF mAb therapy in the treatment of DJD-associated pain in client-owned cats (7).

The objectives of the current study were to extend the proof-of-concept study findings to evaluate the safety and efficacy of frunevetmab frunevetmab (approved as Solensia® in the European Union)^2^ treatment in a multisite field study. This placebo-controlled, randomized study also evaluated two routes of administration for the anti-NGF mAb, included a longer period of assessment, and incorporated accelerometry as an outcome measure.

## Materials and Methods

### Study Design

This study was designed as a multisite, randomized, double-masked, placebo-controlled study with three treatment groups. All cats received two injections, 28 days apart: Group 1 received frunevetmab intravenously (IV) then subcutaneously (SC), Group 2 received frunevetmab SC twice, and Group 3 received vehicle control IV then SC. Outcome measures included changes in owner assessments, objectively measured activity (accelerometry), and veterinary orthopedic examinations. Adverse events were also evaluated. The study was reviewed and approved by the Zoetis Ethical Review Board. Written informed consent was obtained from the owners for the participation of their cats in this study.

### Study Sites and Personnel

This study was conducted at 15 small animal clinics located in the United States. A single licensed veterinarian served as the Investigator at each site, while at least one additional person at the practice served as the Dispenser/Treatment Administrator. The Investigator could serve as an Examining Veterinarian or could designate an additional veterinarian to perform physical and orthopedic examinations. All personnel except the Dispenser/Treatment Administrator were masked to the treatment.

### Study Timeline

**Figure 1:**
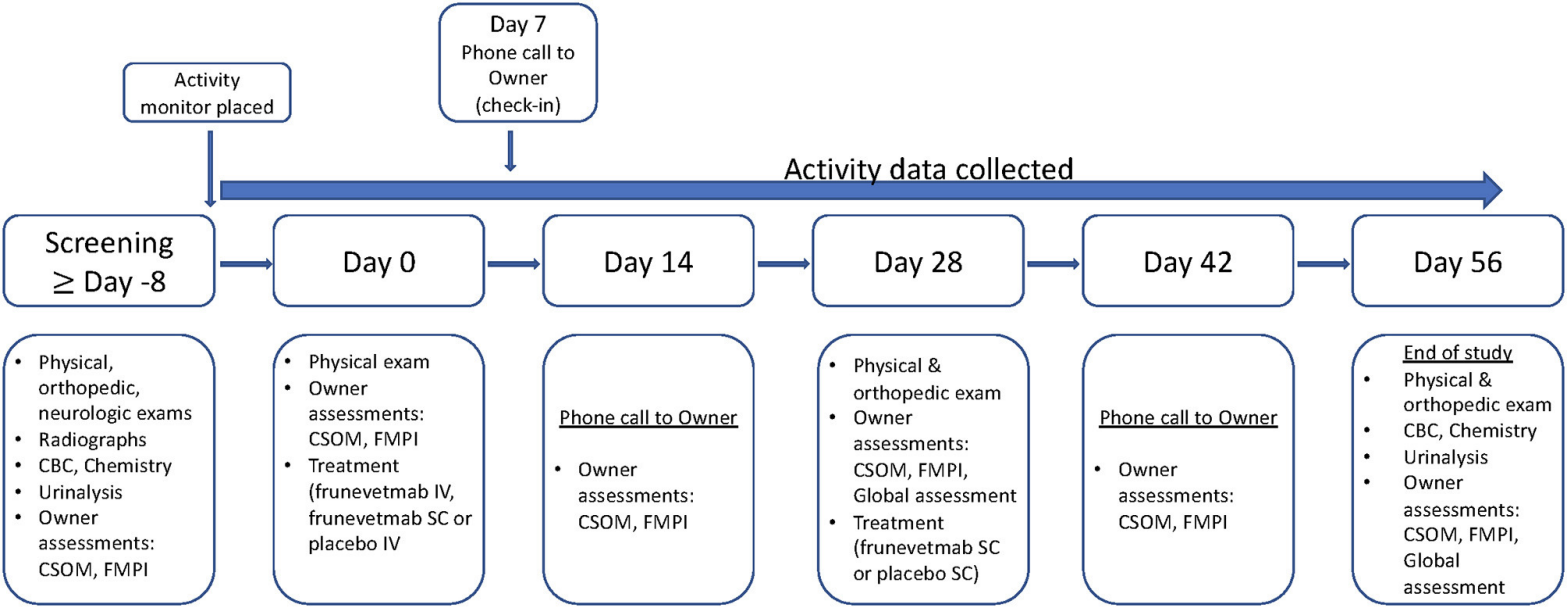
Timeline of events for the pilot field study. CBC, complete blood count; CSOM, client-specific outcome measure; FMPI, feline musculoskeletal pain index.

The study timeline is outlined in Figure 1. Cats were screened (Screening visit) with physical, neurologic, and orthopedic evaluations and lab work (CBC, serum biochemistry, urinalysis); owners completed outcome measures (described in a later section). After Screening, and at least 8 days prior to Day 0, cats had an activity monitor attached to their collar and the subsequent time period was considered baseline activity data. On Day 0, a second physical examination was completed: cats were then injected with either frunevetmab (IV or SC) or placebo (IV); owners completed the clinical metrology instruments (CMIs; see section on outcome measures for details) that were designated as the baseline outcome measures. Owners received a telephone call for a status update plus completion of the CMIs on Day 14 (±2). On Day 28 (±3), cats were evaluated with a physical and orthopedic exam and were then given a second injection of frunevetmab (SC) or placebo (SC); owners completed the CMIs. Owners received a telephone call for a status update and completed the CMIs on Day 42 (±3). On Day 56 (±3), cats returned for a final study visit and were evaluated with a physical and orthopedic exam. Activity monitors were removed at this visit, CMIs were completed by the owners, and samples for lab work were collected.

### Screening Measures and Inclusion/Exclusion Criteria

Cats of any sex and breed were eligible for screening if they were >6 months of age, weighed ≥2.5 kg, lived exclusively indoors, were in generally good health, or had stable chronic conditions (including renal disease with an IRIS classification of stages I and II) as assessed by physical examination, medical history, and laboratory assessments of blood and urine. Cats eligible for enrollment were required to have clinical signs of DJD noted by the owner; pain in at least two joints confirmed by the veterinarian's physical and orthopedic examinations; and radiographic evidence of DJD in at least two of the joints where pain was detected. Cats were also required to have a score of ≥7 on the Client Specific Outcome Measure (CSOM) questionnaire (described under outcome measures).

In addition to failing to meet inclusion criteria, cats were excluded from the study if they were intended for breeding, pregnant, or lactating; had neurologic abnormalities that affected gait; had major surgery within the previous month or planned major elective surgery during the study period; had previous treatment with mAbs except frunevetmab (if given at least 90 days prior to Day 0); or took exclusionary medications. Withdrawal of exclusionary medications was allowed with the following time restrictions: at least 7 days prior to Day 0 for vaccinations, non-steroidal anti-inflammatory medications, and use of amantadine, gabapentin, and tricyclic antidepressants; at least 30 days prior to Day 0 for short-acting steroids; and at least 60 days prior to Day 0 for mid- to long-acting steroids. Joint supplements and diets were permitted if the cats had received them for at least 45 days and would continue to throughout the study period.

### Orthopedic Evaluation

During each orthopedic examination, every limb of the cat was examined and joints were graded for pain, crepitus, effusion, and thickening. Spinal column segments were examined and graded for pain. Scores for pain ranged from 1 to 5—similar to that previously described except that a 1–5 scale was used instead of 0–4 (3); scores for effusion, crepitus, and thickening ranged from 1 to 3 (again similar to that used previously, except that a 1–3 scale was used instead of 0–2). These scores were summed to create a Total Pain Score (sum of individual pain scores) and a Joint Disability Score (sum of crepitus, effusion, and thickening scores).

### Radiographic Evaluation

Radiographs were used to confirm the diagnosis of DJD. Only joints where pain was found during the orthopedic evaluation were radiographed. Radiographic features assessed have been described in detail previously (27, 28). Radiographs were performed only once to ensure eligibility for the study.

### Randomization

Once cats were enrolled into the study, they were randomly assigned to one of three treatment groups: frunevetmab (IV then SC or SC then SC regimens) or placebo (IV then SC). Treatments were administered using unit dosing with cats weighing 2.5–7.0 kg receiving 1 mL of 7 mg/mL frunevetmab injectable solution (or an equivalent volume of vehicle) and cats weighing >7.0–14 kg receiving 2 mL of 7 mg/mL frunevetmab injectable solution (or an equivalent volume of vehicle). This targeted a dosage range of 1.0–2.8 mg/kg. Randomization was based on order of entry into the study at each study site.

### Masking and Administration

To maintain masking, treatments were administered only by the Dispenser/Treatment Administrator at each study site. Owners, examining veterinarians, and other personnel who interacted with owners were not present when treatments were administered. For intravenous treatment administration, the Treatment Administrator could select the most suitable vein. If the study site routinely clipped fur for intravenous injections, then all cats at that site had similar clipping at the time of the 1st treatment to maintain masking. Similarly, the location of subcutaneous treatment administration was at the discretion of each study site's Treatment Administrator based on their site's standards.

### Investigational Product

The investigational veterinary product was frunevetmab (formerly known as NV-02 and manufactured at BioNua, Tullamore, Co., Offaly, Ireland). Frunevetmab is a felinized anti-NGF monoclonal antibody formulated as a sterile solution containing 7 mg/mL of frunevetmab in histidine buffer (10 mM histidine, 5% sorbitol, 0.01% polysorbate, pH 6.0). It was packed as single-use 2-mL vials containing 1.2 mL of solution and was stored at 2–8°C. The placebo control was vehicle (histidine buffer) packaged and stored under the same conditions.

### Efficacy Outcome Measures

The *a priori* primary outcome measure was the owner completed Client Specific Outcome Measures (CSOM). Secondary outcome measures were the Feline Musculoskeletal Pain Index (FMPI), accelerometry data, the owner global assessment, and the veterinarian assessments.

### Clinical Metrology Instruments (CMIs)

Owner-completed questionnaires (CMIs) were used as previously described (3, 29). These included two CMIs previously shown to be responsive in studies of treatments for DJD-associated pain in cats (7, 29, 30). CMIs were completed by a single owner for each cat, and owners were not given access to their prior assessments.

### Client-Specific Outcome Measures (CSOM) Questionnaire

The CSOM queries owners about their cat's ability to perform three individually tailored activities on a scale from “No problem” to “Impossible.” The CSOM used in this study was constructed as previously described (7, 29, 30) (https://cvm.ncsu.edu/research/labs/clinical-sciences/comparative-pain-research/clinical-metrology-instruments/). Activities were selected for each cat/owner dyad at the Screening visit. Owners were allowed to change one, two, or all activities after having observed their cat during the period prior to Day 0; once cats received their first treatment on Day 0, activities could no longer be changed. Ratings were converted to numerical scores from 1 (No problem) to 5 (Impossible) and summed, giving a total possible score range of 3–15. A reduction from the Day 0 score of ≥2 was defined as a treatment success; number of successes/failures and changes in total scores from Day 0 were compared between groups for Days 14, 28, 42, and 56.

### Feline Musculoskeletal Pain Index (FMPI)

The FMPI used was based on previous work (7, 29) with additional questions as described below. The FMPI asks owners to rate their cat's ability to perform a set of 14 activities on a Likert scale from normal to most impaired, with an option to select “Not applicable” for questions related to stairs and interactions with other pets. Three questions (15–17) asked owners to rate their cat's amount of pain, quality of life, and happiness using the same Likert scale (best to worst). Owner ratings for Questions 1–17 were converted to numerical scores from 1 (normal) to 5 (most impaired/worst) and summed across the activities for a total possible score range of 17 (if all questions were answered) to 85. Scores were expressed as both total score and as a percentage of total possible score as previously described (7, 29) to account for questions where owners were unable to answer. A reduction from the Day 0 score of ≥10 was defined as a treatment success [criteria established in (7)]; number of successes/failures and changes in scores for total score and percent possible scores were compared between groups for Days 14, 28, 42, and 56.

Four additional “Yes/No” questions (Questions 18–21) not previously included were incorporated in the FMPI following the Likert scale activities; these asked about specific cat behaviors or features. The number and percentage of yes/no responses were summarized by study day and treatment group; Day 0 scores were compared to Days 14, 28, 42, and 56.

### Accelerometry (Activity)

Once enrolled, cats were fitted with an activity monitor (AM; Actical, Starr Life Sciences Company, Oakmont, PA, USA) on a collar or harness. These monitors—attached to collars or harnesses—have been validated as a measure of distance moved in cats (31) and have been used in previous studies of therapeutics for relief of DJD pain in cats (7, 29, 30, 32). The activity monitor was set up to gather a minimum of 7 days of baseline activity data and then to record continuously throughout the study. On Day 56 (or after any early withdrawal), data were downloaded as activity counts per minute for each minute of the study duration. These were then averaged across each week of the study, and the changes from baseline in weekly average per-minute activity counts were compared between groups.

### Owner Global Assessment

On Days 28 and 56, owners were asked to make a global assessment of the treatment's success in controlling clinical signs of DJD in his/her cat. These were scored as the following: Excellent (clinical signs eliminated or reduced to an inconsequential level), Good (clinical signs at least 50% reduced), Fair (clinical signs <50% reduced), or Poor (clinical signs unaffected by treatment). Treatment success was defined as a rating of Good or Excellent; the number and percentage of treatment success/failures were compared between groups at Days 28 and 56.

### Veterinary Assessments

Orthopedic examinations were performed at Day 28 and Day 56 as described. These were to be conducted by the same veterinarian at a given site for each timepoint. Summary scores were calculated for total pain score (Total Pain) and joint disability (Joint Disability; sum of crepitus, effusion, and thickening). These scores were evaluated for the change from Screening to Day 28 and Day 56 and compared between groups.

### Safety Outcome Measures

Safety assessments were made based on the findings of physical and neurological examinations (Screening, Day 28, and Day 56); injection site evaluations (Day 0, Day 28, and Day 56); clinical pathology at Screening and on Day 56 (serum biochemistry, CBC, urinalysis); and adverse events reported by owners. All samples were processed at a central laboratory (IDEXX Laboratories Inc. West Sacramento, CA, USA). Values were compared for Screening and Day 56. Descriptions of adverse events were collected from owners throughout the study, coded based on the Veterinary Dictionary for Drug Regulatory Activities (VeDDRA) reporting system; numbers of adverse events were compared between groups.

### Removal From the Study

Cats could be removed from the study at any time if, in the opinion of the veterinarian or owner, the cat was experiencing excessive discomfort, injury, or illness, or if owners were non-compliant with the protocol. Owners could elect to withdraw their cat from the study at any time without penalty. Cats who were withdrawn early for reasons unrelated to treatment and cats with significant study deviations were not included in the efficacy evaluation; all cats were included in the safety analysis. Cats who were withdrawn due to owners' perceived lack of efficacy were considered treatment failures for all subsequent evaluation days.

### Statistical Analysis

Groups were compared for distribution of age and weight using *t*-tests; sex distribution was compared using chi-square analysis.

Treatment success/failure analyses for CSOM, FMPI, and Owner global assessment were evaluated using appropriate methods for binary outcomes (GLIMMIX procedure; SAS version 9.4; Cary NC, USA) assuming a binomial distribution and logit link. The model included treatment group as a fixed effect with site and treatment group by site interaction as random effects. Fisher's exact (two-tailed) test was applied to the four yes/no questions of the FMPI for each study day, and to Question 16 (quality of life) on Days 42 and 56. Success/failure rates were used to calculate values for number needed to treat (NNT) for CSOM.

For each continuous efficacy variable, repeated-measure analysis of covariance (ANCOVA) modeling was used to test for possible differences between treatment groups for the changes from Day 0/Screening to each subsequent study day. Pairwise comparisons to placebo were derived from the model. Mean and standard deviations for outcome measures across placebo and frunevetmab treatment groups were used to calculate standardized effect sizes of treatment over placebo (ES) for CSOM.

Weekly average per-minute activity counts were evaluated by RMANCOVA (MIXED procedure). The percent change on a weekly basis from baseline and percent change within a dosing period were also evaluated. The percent of cats with a <-10, −5, 0, 5, or 9% improvement in average per-minute activity counts (weekly or by dosing period) vs. baseline was evaluated by Fisher's exact test (FREQ procedure) on each evaluation week or treatment period. Cats with <4 days of activity monitor data for any given week were excluded for that week. Testing was two-sided at the significance level *p* = 0.05.

## Results

### Subjects

Details of the numbers of cats included in efficacy and safety analyses are given in the CONSORT diagram (Supplementary Figure 1).

A total of 126 cats were randomized to treatment groups. Demographic characteristics of each group of cats enrolled in the study are shown in Table 1. There were no significant differences between the groups for age, weight (*p* = 0.756), or sex distribution (*p* = 0.442). Nine of 85 frunevetmab-treated cats and three of 41 placebo-treated cats did not complete the study. Reasons for discontinuation included “Adverse Event” for three (7.1%) frunevetmab:IV/SC-treated cats, “Owner perceived lack of therapeutic effect” for one (2.3%) frunevetmab:SC/SC-treated cat and one (2.4%) placebo-treated cat, “Owner withdrew consent” for two (4.8%) frunevetmab:IV/SC-treated cats, “Protocol deviation” for three (7.0%) frunevetmab:SC/SC-treated cats and one (2.4%) placebo treated cat, and “Other” for one (2.4%) placebo-treated cat. Missing/invalid or excluded data points for any one efficacy measure did not exclude an individual from all assessments; details of the study population included in each of the analyses are provided in Supplementary Tables 1A,B.

**Table 1:** Demographic characteristics of cats in each treatment group.

**Parameter**		**Placebo**	**Frunevetmab 1.0–2.8 mg/kg**		***p*-value**
			**Day 0 IV, Day 28 SC**	**Day 0 SC, Day 28 SC**	
Number of cats		41	42	43	N/A
Breed	DSH DMH DLH Ragdoll Siamese All other purebreeds^*^ Other mixes	28 (68.3%) 2 (4.9%) 4 (9.8%) 1 (2.4%) 1 (2.4%) 3 (7.3%) 2 (4.9%)	29 (69.0%) 3 (7.1%) 3 (7.1%) 1 (2.4%) 0 (0%) 4 (9.5%) 2 (4.8%)	28 (65.1%) 3 (7.0%) 2 (4.7%) 1 (2.3%) 2 (4.7%) 6 (14.0%) 1 (2.3%)	N/A
Age (years)	Median (Min:Max)	13.83 (3.0:9.2)	12.38 (3.2:10.5)	12.58 (3.3:8.9)	
Weight (kg)	Median	5.40	5.45	5.80	*p* = 0.756
Sex	Female spayed Male castrated	25 (61.0%) 16 (39.0%)	27 (64.3%) 15 (35.7%)	22 (51.2%) 21 (48.8%)	Chi-sq. 1.636; *p* = 0.441
Total orthopedic pain score	Median (Min:Max)	30.5 (21.0:49.0)	29.5 (19.0:77.0)	31.0 (21.0:48.0)	*p* = 0.152
Total joint debility score	Median (Min:Max)	52.0 (40.0:95.0)	52.0 (37.0:81.0)	52.0 (36.0:66.0)	*p* = 0.845

*DLH, domestic longhair (non-pedigree); DMH, domestic medium hair (non-pedigree); DSH, domestic short hair*.

*N/A, Not applicable*.

* *Purebreeds included American Shorthair, Angora, Bombay, British Shorthair, Devon, Maine Coon Cat, Manx, Munchkin, Norwegian Forest Cat, Oriental, Persian*.

### Efficacy

Statistical comparison showed no meaningful difference between the two frunevetmab-treated groups (using repeated-measure analysis of variance); therefore, they were combined for analyses of most efficacy variables, with the exception of the CSOM, where group differences were found for some timepoints, and are also reported.

### CSOM

With respect to success/failure designation, significantly more cats were considered treatment successes based on CSOM assessment in the combined frunevetmab-treated groups (76.1 and 80.3%) compared to placebo (55.3 and 44.7%) on Days 42 (*p* = 0.0479) and 56 (*p* = 0.0033), but not on Days 14 and 28 (Table 2). The NNT for frunevetmab at Day 42 was 5 and at Day 56 was 3.

**Table 2:** CSOM success rates for each treatment group and each evaluation time point.

**Study day**	**Placebo**	**Frunevetmab 1.0–2.8 mg/kg**	***p*-value**
Day 14	21/35 (60.0%)	45/73 (61.6%)	0.850
Day 28	20/38 (52.6%)	52/74 (70.3%)	0.101
Day 42	21/38 (55.3%)	54/71 (76.1%)	**0.048**
Day 56	17/38 (44.7%)	57/71 (80.3%)	**0.003**

*Shown are the numbers of cats that were a treatment success/total numbers of cats, with the percentage of cats which were a treatment success, shown in parentheses*.

*Significant p-values are denoted in bold*.

The overall treatment effect for the change from pretreatment in total CSOM scores was statistically significant for the combined frunevetmab-treated group (*p* = 0.012). For the median change from pretreatment total CSOM scores (Table 3), statistically significant differences were found between the frunevetmab-treated group and the placebo-treated group at Days 42 (*p* = 0.0078) and 56 (*p* < 0.0001). Separating the frunevetmab treatment groups, a larger median decrease was seen in the frunevetmab:SC/SC group (Table 3). Statistically significant differences were found between the frunevetmab:SC/SC-treated group and the placebo-treated group from Day 28 onward (*p* ≤ 0.034; Table 3). Mean changes in score were used to calculate effect size (ES); the ES was 0.45 and 0.65 at Day 42 and Day 56, respectively.

**Table 3:** CSOM scores, mean (SEM), and median (range) at each time point for each group, and the median changes in CSOM scores for each group at each time point compared to Day 0.

**Study day**	**Group**	**Number of animals**	**Mean total CSOM score (SEM)**	**Change in mean total CSOM score vs. Day 0 (SEM)**	**Median total CSOM score (min max)**	**Change in median total CSOM score vs. Day 0**	***p*-value**
0	Placebo	40	10.45 (0.29)	–	10 (7–15)	N/A	0.711^*^
	Frunevetmab (combined)	85	10.35 (0.21)	–	10 (7–15)	N/A	
	Frunevetmab IV/SC	42	10.33 (0.30)	–	10 (7–15)	N/A	0.960
	Frunevetmab SC/SC	43	10.37 (0.30)	–	10 (7–14)	N/A	
14	Placebo	35	8.06 (0.43)	−2.31 (0.39)	8 (3–15)	−2	0.359
	Frunevetmab (combined)	73	7.71 (0.28)	−2.52 (0.28)	7 (3–14)	−3	
	Frunevetmab IV/SC	36	7.83 (0.44)	−2.58 (0.40)	8 (4–13)	−3	0.517
	Frunevetmab SC/SC	37	7.59 (0.37)	−2.46 (0.41)	7 (3–14)	−3	0.354
28	Placebo	38	7.95 (0.44)	−2.37 (0.44)	8 (3–13)	−2	0.055
	Frunevetmab (combined)	73	6.93 (0.30)	−3.29 (0.32)	7 (3–14)	−3	
	Frunevetmab IV/SC	36	7.28 (0.43)	−3.11 (0.44)	7.5 (3–13)	−3	0.229
	Frunevetmab SC/SC	37	6.59 (0.43)	−3.46 (0.46)	6 (3–14)	−4	**0.034**
42	Placebo	38	7.84 (0.44)	−2.55 (0.45)	8 (3–14)	−2	**0.008**
	Frunevetmab (combined)	70	6.49 (0.33)	−3.83 (0.34)	6 (3–14)	−4	
	Frunevetmab IV/SC	34	7.06 (0.48)	−3.32 (0.48)	6.5 (3–14)	−3	0.133
	Frunevetmab SC/SC	36	5.94 (0.43)	−4.31 (0.48)	5.5 (3–12)	−5	**0.002**
56	Placebo	37	8.05 (0.53)	−2.32 (0.51)	9 (3–15)	−1	**<0.0001**
	Frunevetmab (combined)	70	5.99 (0.32)	−4.26 (0.34)	6 (3–15)	−4	
	Frunevetmab IV/SC	33	6.58 (0.47)	−3.76 (0.47)	6 (3–15)	−4	**0.011**
	Frunevetmab SC/SC	37	5.46 (0.42)	−4.70 (0.48)	5 (3–12)	−5	**<0.0001**

* *Day 0 p-value was generated by analysis of variance with Treatment as Fixed effect with Site and Treatment by Site as Random effects*.

*N/A, Not applicable*.

*Significant p-values denoted in bold. p-values refer to the comparsion of the respective treatment group to the placebo group*.

### FMPI

#### Questions 1–17

There were significantly more cats with treatment success (reduction in score of ≥10) in the frunevetmab-treated group (69.6 and 67.1%) compared to the placebo-treated group (32.4 and 40.5%) for Day 42 (*p* = 0.0076) and Day 56 (*p* = 0.024). The overall treatment effect in the change in the median percent of maximum possible total FMPI scores compared to Day 0 was statistically significant (*p* = 0.032). Statistically significant differences were found between the frunevetmab-treated and placebo-treated groups at Day 42 (*p* = 0.0002) and Day 56 (*p* = 0.016).

Because of the recent increasing interest in measuring overall quality of life, responses to Question 16 (owner rating of quality of life) were analyzed separately. In the combined frunevetmab-treated group, 37.0 and 39.5% of the cats were rated as having excellent quality of life on Days 42 and 56, respectively, compared to 7.9 and 15.8% of the placebo-treated cats (*p* ≤ 0.025).

#### Questions 18–21

No statistically significant differences were found between groups on any study day for questions 18 and 19, which concerned ease of movement and whether single or multiple small jumps were required for jumping up. Frunevetmab-treated cats had significantly better responses (*p* < 0.05) on Day 56 for question 20 (which concerned hitting legs on an object when jumping up) and on Day 42 for question 21 (which concerned reaching down to shorten a jump down).

### Accelerometry

**Figure 2:**
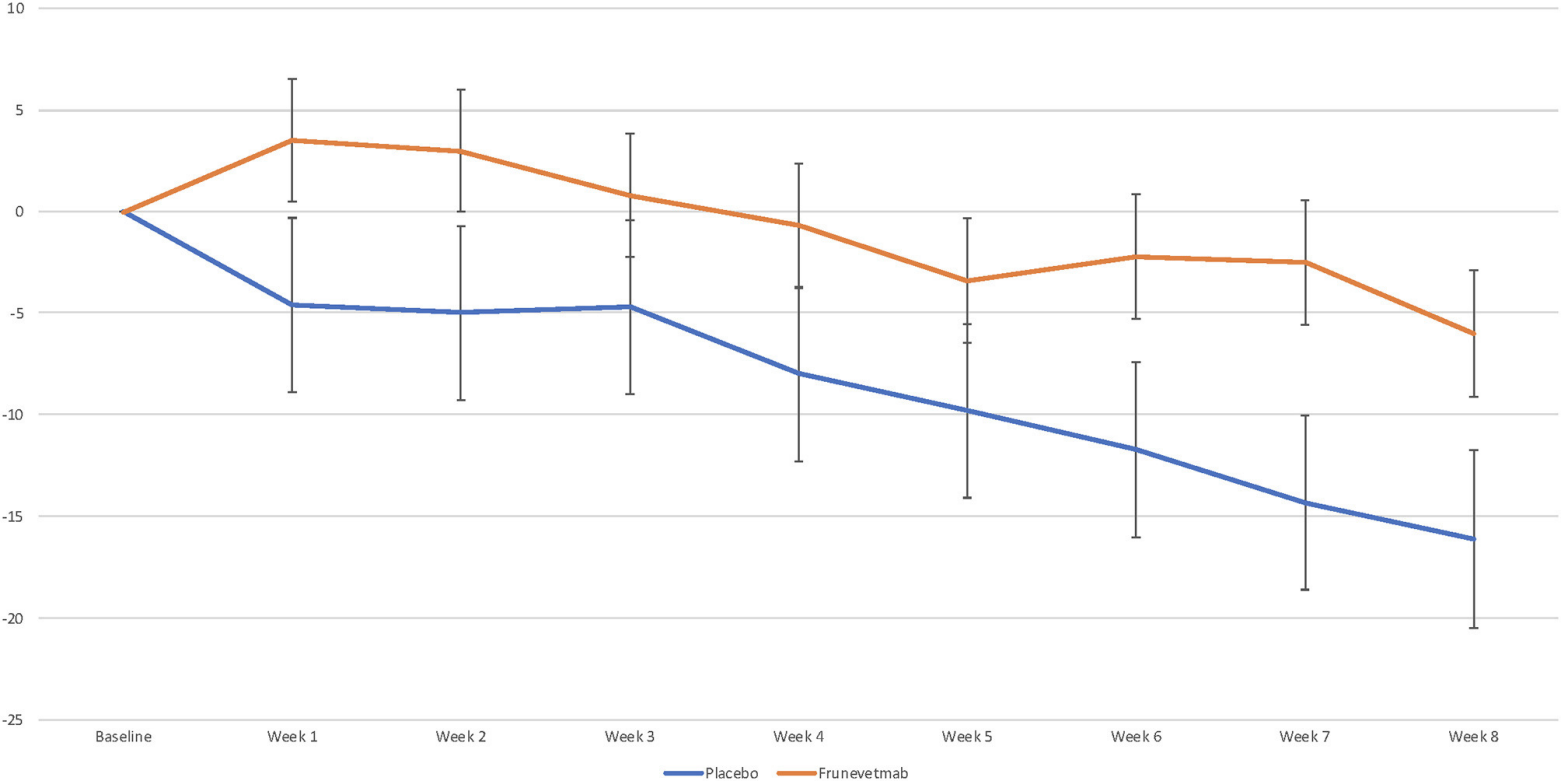
Mean percentage change vs. pretreatment in mean per-minute activity averaged over each week (± SEM).

Weekly average per-minute activity counts measured by activity monitors decreased for both treatment groups compared with pretreatment baseline activity (Figure 2). The analysis of weekly activity count data with baseline as a covariate showed that neither the week by treatment interaction nor the main effect of treatment was significant (*p* > 0.05). For weekly percentage change from baseline, the week by treatment interaction was not significant and the main treatment effect *p*-value was 0.0637. When period 1 (Weeks 1–4) and period 2 (Weeks 5–8) were evaluated, the percentage change from baseline was not significantly different between the groups (*p* = 0.129; −5.6% ± 3.9 for placebo, and 1.6% ± 2.74 for frunevetmab) in period 1, but neared significance in period 2 (*p* = 0.0595; −12.5% ± 3.9 for placebo, and −3.4% ± 2.79 for frunevetmab).

Across the entire 8-week data collection period post-treatment, the frunevetmab-treated group showed little overall change from baseline (mean decrease 0.9%) while the placebo-treated group showed a mean of 9.3% decrease in weekly activity. When percent success based on the magnitude of change from baseline was examined, no differences were detected between treatment groups at a cutoff point of ≥5%. At a cutoff point of 0%, the frunevetmab-treated group had significantly higher success rates (*p* < 0.05) compared to the placebo-treated group at Week 1 (51 vs. 31%) and Week 2 (54 vs. 32%).

Given that both groups showed an overall decrease in activity (further detail in the discussion), which appeared to be mitigated by frunevetmab, success rates for negative cutoffs were evaluated; cats whose activity did not decrease by more than a set cutoff were considered a success. At a cutoff point of −5%, the frunevetmab-treated group had significantly higher success rates (*p* < 0.05) compared to the placebo-treatment group at Week 2 (69 vs. 43%) and Treatment Period 1 (62 vs. 39%). At a cutoff point of −10%, the frunevetmab-treated group had significantly higher success rates (*p* < 0.05) compared to the placebo-treated group at Week 1 (77 vs. 58%), Week 2 (80 vs. 51%) and Week 3 (69 vs. 41%), Week 5 (60 vs. 32%), Week 7 (55 vs. 33%), and Treatment Period 1 (71 vs. 47%).

### Owner's Global Assessment

Significantly more cats were considered treatment successes on the owner's global assessments (Excellent or Good response) in the frunevetmab-treated group (55.4 and 72.9%) compared to the placebo-treated group (26.3 and 32.4%) on Day 28 (*p* = 0.0134) and Day 56 (*p* = 0.0030) (Table 4).

**Table 4:** Number (percent) success for owner global assessment in each treatment group.

**Study day**	**Placebo**	**Frunevetmab**	***p*-value**
Day 28	10/38 (26.3%)	41/74 (55.4%)	**0.013**
Day 56	12/37 (32.4%)	51/70 (72.9%)	**0.003**

*Significant p-values denoted in bold*.

### Veterinary Orthopedic Examination

A summary of the orthopedic pain scores is shown in Table 1. For the Total Pain score at Screening, the mean score was 32.25 (median 30.50) in the placebo-treated group and 31.65 (median 31.00) in the combined frunevetmab-treated groups. For the Total Joint Debility score at Screening, the mean score was 53.88 (median 52.00) in the placebo-treated group and 53.58 (median 52.00) in the combined frunevetmab-treated groups.

There were no significant differences between treatment groups for the change in Total Pain score or the Total Joint Debility score on Days 28 or 56.

### Safety

Six cats total were withdrawn from the study for reasons associated with safety. Three cats were withdrawn due to adverse dermatological effects in the neck region associated with the use of collars (used to carry the activity monitors). Three cats were withdrawn for reasons related to other adverse events. Withdrawals are summarized in Supplementary Table 2.

There were 122 adverse events described in 67 cats; 119 of these were categorized as non-serious. These were reported in 30 (71.4%) cats in group 1 (frunevetmab given IV then SC), 21 (48.8%) cats in group 2 (frunevetmab given SC twice), and 16 (39%) cats treated with placebo. The abnormal health events were generally reported in similar frequencies in the placebo-treated and frunevetmab-treated groups (Table 5). Emesis, renal insufficiency, and dermatitis/eczema were the most frequently reported abnormal health events across all groups. The incidence of emesis was higher in the placebo-treated cats (14.6%) compared to the frunevetmab-treated cats (7.1% in IV/SC group and 7.0% in SC/SC group). The percentage of cats with renal insufficiency events was higher in frunevetmab-treated cats (2.4% in IV/SC group and 9.3% in SC/SC group) compared to placebo (0%). Most of the skin-related health events were associated with the collars that were worn by all cats to mount the activity monitor. However, skin-related abnormal health events appeared to be reported in increased frequencies in the two frunevetmab-treated groups (dermatitis/eczema in 23.8% of the cats in the IV/SC group and in 14.0% in the SC/SC group, compared to 2.4% in the placebo-treated group, respectively). No injection site reactions were noted in any cat.

**Table 5:** Adverse events reported in two or more cats in any treatment group.

		**Placebo**	**Frunevetmab**	
System organ	Adverse effect	Day 0: IV Day 28: SC	Day 0: IV Day 28: SC	Day 0: SC Day 28: SC
Digestive tract	Diarrhea	0/41 (0.0%)	3/42 (7.1%)	1/43 (2.3%)
	Emesis	6/41 (14.6%)	3/42 (7.1%)	3/43 (7.0%)
	Gingival disorder	3/41 (7.3%)	1/42 (2.4%)	1/43 (2.3%)
	Tooth disorder	3/41 (7.3%)	0/42 (0.0%)	0/43 (0.0%)
Eyes	Eye disorder (NOS)	2/41 (4.9%)	1/42 (2.4%)	1/43 (2.3%)
Musculoskeletal	Lameness	0/41 (0.0%)	2/42 (4.8%)	1/43 (2.3%)
Renal/urinary	Renal insufficiency	0/41 (0.0%)	1/42 (2.4%)	4/43 (9.3%)^†^
	Urine abnormalities	2/41 (4.9%)	1/42 (2.4%)	2/43 (4.7%)
Respiratory tract	Cough	1/41 (2.4%)	2/42 (4.8%)	0/43 (0.0%)
	Rhinitis	2/41 (4.9%)	0/42 (0.0%)	0/43 (0.0%)
	Sneezing	0/41 (0.0%)	3/42 (7.1%)	1/43 (2.3%)
Skin and appendages	Alopecia	0/41 (0.0%)	3/42 (7.1%)	1/43 (2.3%)
	Dermatitis and eczema^*^	1/41 (2.4%)	10/42 (23.8%)	6/43 (14.0%)
Systemic	Lethargy	1/41 (2.4%)	2/42 (4.8%)	1/43 (2.3%)

*Shown are the numbers of cats with the adverse event/total numbers of cats in that group, with percentage of cats affected in parentheses*.

*NOS, not otherwise specified*.

† *Details of case in the IV/SC group: Case 16-15: enrolled without chronic kidney disease; increased SDMA at Day 56; no other changes in renal parameters—insufficient data for IRIS staging*.

*Details of cases in the SC/SC group: Case 16-07: enrolled at IRIS stage 2; increased SDMA and serum creatinine at Day 56; remained IRIS stage 2. Case 04-15: enrolled at IRIS stage 2; increased SDMA associated with a single episode of dehydration at Day 28; resolved with a single fluid treatment; SDMA within the normal range at Day 56; remained at IRIS stage 2. Case 16-09: enrolled at IRIS stage 2; increased SDMA and serum creatinine associated with a urinary tract infection (UTI) diagnosed at Day 56; remained at IRIS stage 2. Case 06-09: enrolled at IRIS stage 2; increased SDMA and serum creatinine at Day 56; increased to IRIS stage 3 (this case was captured as two separate adverse events)*.

* *Areas affected by dermatitis/eczema were as follows: placebo, face/neck (n = 1); IV/SC frunevetmab, face/neck (n = 9), location not specified (n = 1), SC/SC frunevetmab, face/neck (n = 4), location not specified (n = 1), intradigital (n = 1)*.

Three cats (one frunevetmab-treated cat [IV/SC] and two placebo-treated cats) had serious adverse health events, including two cats who died during the study (one IV/SC frunevetmab-treated cat and one placebo-treated cat). The frunevetmab-treated cat with preexisting anemia was diagnosed with acute pancreatitis, immune-mediated hemolytic anemia, and thrombocytopenia on Day 16 and died on Day 31 in spite of treatment. One placebo-treated cat died suddenly on Day 33; the death was attributed to endomyocarditis based on necropsy findings. The third cat with an adverse event classified as serious (placebo-treated group) was reported to be reluctant to move on Day 35 and recovered by Day 56 following treatment with potentiated sulfonamide. The attending veterinarian considered the condition likely due to the OA and associated pain.

No clinically relevant differences were observed in the clinical biochemistry and hematology parameters between groups. Selected serum biochemistry (hepatic, renal) and urinalysis results at Screening and study exit (Day 56) are provided in Supplementary Table 3. Frunevetmab was used without incident in conjunction with various medications including, but not limited to, antimicrobials, parasiticides, and nutritional supplements.

## Discussion

In this study, frunevetmab administered twice with a 28-day interval at a dose range of 1.0–2.8 mg/kg by IV or SC injection demonstrated statistically significant efficacy to placebo in the control of pain associated with DJD in cats. Efficacy was found for both percentage successes and median change from pretreatment values for two different owner assessments despite the documented high placebo effect in chronic pain trials in cats (33) and relatively small population size. Previous placebo-controlled studies in cats have demonstrated placebo effects as high as 74% success among placebo-treated cats (33); this has made demonstration of significant treatment effects difficult. Efficacy data in this study were further supported by objective accelerometry and by the owner global assessment. In addition to the statistical significance, changes were clinically relevant as shown by effect size (ES) and numbers needed to treat (NNT).

Statistically significant endpoints are important for the success or failure of a study, but it is important to understand the size, or clinical meaning, of the treatment effects. Given that a total CSOM score of 3 in this study equated to “normal,” and the median initial score was 10, then the maximum change possible in this study was, on average, 7 (a decrease in disability from 10 to 3). Therefore, with treatment success defined, *a priori*, as a change of “2,” this equated to a 29% reduction in disability [(2/7)^*^100], considered clinically meaningful in human medicine (34, 35). In the present study, the median decrease in disability scores in the treatment group was “4” at Days 42 and 56 (a 57% reduction in disability). In human medicine, a 50% reduction in pain and disability is considered “very much improved” (34, 35). Similar calculations for the FMPI indicate that a change of 10 points (used as the success/failure cutoff) represents a 26.3% reduction in pain and disability—again, considered clinically meaningful. We can therefore conclude that the criteria for success were clinically meaningful, as was the reduction in clinical signs seen.

Effect sizes are another way to assess the magnitude or strength of the findings from research studies of different designs with varied endpoints. Standardized effect size evaluates the differences between groups using the means of each group and the population standard deviation. This unitless measure allows comparison across studies and an estimation of the relative size of the effect, separate from just statistical significance. The effect size for anti-NGF mAbs in humans has varied, depending on the dose, from −0.15 to 0.7 (21). The effect size for NSAIDs in hip and knee OA in humans has been found to be ~0.3 when based on high-quality trials (36, 37). Frunevetmab in this study (ES 0.45 at day 42 and 0.65 at day 56) clearly showed an effect size greater than NSAIDs in humans, and comparable to the best effect size for anti-NGF mAbs reported in humans. This means that over 60% of the control group was below the mean of the frunevetmab group after 42 days, and over 70% were below the mean after 56 days. The ES reported here is similar to the ES reported in the early proof-of-concept work with this mAb, frunevetmab (0.74 at 21 days following treatment) (7), further supporting the conclusions of efficacy. In early proof-of-concept work in dogs, a canine anti-NGF mAb (ranevetmab) was reported to have effect sizes of 0.38–0.96 across different owner assessments at 28 days following treatment (25). Another accepted way of defining clinical utility of a therapeutic is numbers needed to treat (NNT) where lower numbers indicate greater effectiveness. For frunevetmab, the NNT at Day 42 was 5 and at Day 56 the NNT was 3. These values compare extremely favorably with the NNT for NSAIDs in humans [between 3 and 13 depending on the criteria for success (38)]. There are no published data from studies of chronic pain in cats (or dogs) with which to compare these values. However, using data described in early proof-of-concept work with frunevetmab, the NNT at 21 and 42 days after treatment were 3.8 and 3.7, respectively (7), again supporting the current results. The adjunct global assessment is valuable, as it may encompass several dimensions known to be affected by chronic pain (39), including behavioral and affective/emotional domains (40, 41); however, its accuracy as a solitary predictor of success is uncharacterized.

While the accelerometry data showed a difference between the frunevetmab and placebo groups, the change in activity over time was different from what might be anticipated. Cats receiving frunevetmab showed little change in their measured activity while the cats receiving placebo had a significant decrease in activity from baseline. In most previous studies, efficacy has been measured as an increase in activity relative to baseline (7, 29, 30, 42). The exception to this is a study that evaluated gabapentin for the treatment of OA-associated pain in cats (4). In that study, cats were significantly less active when receiving gabapentin as compared to placebo; this finding was attributed to a possible sedating effect of gabapentin. However, there are placebo-controlled studies where the placebo group showed an overall decrease from baseline—a decrease that was mitigated by the active treatment (43). Possible explanations for the findings in the current study include a “falsely elevated” baseline or a change in behaviors that is not reflected in overall activity. A falsely elevated baseline could be due to the placement of collars on cats who were not acclimated to them, resulting in excessive scratching at the collars, which would register as activity counts (30, 31). Another possible explanation for a falsely elevated baseline could be due to residual effects of the Screening visit (examination, manipulation, sedation, radiography). Another explanation could be the extra attention paid to the cat by owners during the baseline period as the clinical trial started.

Alternatively (or perhaps in combination), total activity may not be the most appropriate outcome measure; the beneficial effects of treatment with frunevetmab may be best characterized by changes in the *ability* to perform activities, such as jumping—which should be reflected in the owner assessments and would not be reflected in changes in activity counts (44). Furthermore, elevations in intensity of activity would not change activity counts, for instance a cat that runs *instead* of walks or one that climbs and descends the stairs *instead* of simply walking. Additionally, this study was the first to use accelerometry in a multisite field study with cats; while this study demonstrated the feasibility of this measure in indoor cats, the results will need to be evaluated through similar studies in the future.

Even with these limitations, it is still appropriate to compare the two groups for activity. Overall, there is a clear visual separation of the combined frunevetmab-treated group from the placebo-treated group and the main treatment effect approached significance (*p* = 0.0637). The numerical difference in activity between groups was very similar to the proof-of-concept study (~10%) (7). Using a cutoff of minus 10% (meaning a decrease in activity of <10%) to compare the two groups in terms of success/failure, a significant difference between the groups was seen at 1, 2, and 3 weeks after the first injection and 1 and 3 weeks after the second injection.

The difference between placebo and treatment for the change from baseline in activity seen in this study (~8.5% pooled across weeks) is at the upper end of, or greater than, that seen in other studies of cats using NSAIDs. In a study using meloxicam (0.035 mg/kg daily for 3 weeks), the difference in total activity between placebo and meloxicam was 3.22% (29). In a placebo-controlled study of robenacoxib (1 mg/kg daily for 3 or 6 weeks), the difference in activity between the groups was 5% (in favor of robenacoxib) for total activity (https://www.ema.europa.eu/en/documents/variation-report/onsior-v-c-127-ii-0018-g-epar-assessment-report-variation_en.pdf).

For the veterinarian's orthopedic evaluation, there were no significant differences between treatment groups at Day 28 or Day 56. This is not surprising. Our unpublished data (masked for review) shows that veterinarian assessments of chronic pain in cats are insensitive. In contrast to owners who are assessing their pet over a period of time in their habitual environment, veterinarians are attempting to score brief observations of an animal that is in an unfamiliar environment and suffering from stress (45).

Frunevetmab appeared to be very well-tolerated by cats in the study which had a mean age of 12–13 years. The majority of adverse events were digestive issues (similar in frequency between placebo and treated groups) and skin and appendage disorders (most of which were associated with the collar). The frequency of these skin and appendage disorders was much higher in the frunevetmab-treated cats and deserves further research to understand a possible connection. Importantly, skin conditions responded to standard therapeutics, there were no clinically significant differences between the groups with respect to clinical pathology results, and no injection site abnormalities were found for any group. Although variations in clinical renal disease and biochemical values were categorized broadly as renal insufficiency to identify any potential risk of renal effects, these were not necessarily indicative of advancement, or identification, of specific renal disease; case details are shown in Table 5. Based on blood chemistry at the screening visit, 3/85 cats in the treatment group and 1/41 in the placebo group were enrolled with normal serum creatinine values and elevated symmetric dimethylarginine (SDMA) values (>14) consistent with IRIS Stage 1. Almost half of the cats in the treatment and placebo groups, 41/85 and 19/41, respectively, were enrolled with elevated serum creatinine concentrations consistent with IRIS Stage 2 (1.6–2.8 mg/dL). Among cats in the SC/SC group who entered the study in IRIS stage 1 or 2, three had increases in either SDMA, serum creatinine, or both while remaining at IRIS stage 2. One case increased from IRIS stage 2 at enrollment to IRIS stage 3. One cat from the IV/SC group with no preexisting renal condition had mildly increased SDMA at Day 56 (with no other changes in renal parameters). Although there was a higher frequency of renal insufficiency events in the frunevetmab groups, analysis of SDMA, serum creatinine, and blood urea nitrogen did not reveal significant differences between treated groups compared to placebo at the end of the study. While safety remains necessary to evaluate in a larger trial, the results of this study indicate a positive safety profile for frunevetmab.

## Conclusions

Results of this study support the use of frunevetmab for the treatment of chronic pain in cats. As an injectable therapy providing long-lasting effectiveness, frunevetmab would preclude the need to medicate cats orally, which can be difficult for many owners, cats, and the human–animal bond. Its mechanism of action, targeting a major player in the development of maladaptive pain and sensitization, makes it a novel therapy with the potential to dramatically increase the treatment of DJD pain in cats.

## Data Availability Statement

The raw data supporting the conclusions of this article will be made available by the authors, without undue reservation.

## Ethics Statement

The animal study was reviewed and approved by Zoetis Ethical Review Board. Written informed consent was obtained from the owners for the participation of their animals in this study.

## Author Contributions

MG and BDXL participated in the design of the study. BDXL contributed to the statistical analysis. MG, BDXL, and JM all participated in the interpretation of the data, drafting, and revision of the manuscript. All the authors endorsed the content of the work.

## Conflict of Interest

MG and BDXL are paid consultants and have conducted sponsored CE for Zoetis; JAEM is an employee of Zoetis. The authors declare that this study received funding from NexVet, now Zoetis. The funder was involved in the study design, execution and data analysis and publication decision. Data interpretation and writing of this article was performed in part by a Zoetis employee.

## Footnotes

^1^Committee for Medicinal Products for Veterinary Use. 2020. EMA Librela—Summary of Opinion. EMA/CVMP/438128/2020. Available online at: https://www.ema.europa.eu/en/medicines/veterinary/EPAR/librela.

^2^Committee for Medicinal Products for Veterinary Use. 2020. EMA Solensia— Summary of Opinion. EMA/CVMP/631469/2020. Available online at: http://www.ema.europa.eu/en/medicines/veterinary/summaries-opinion/solensia.
