# Supplementary figures and images for: Efficacy and Safety of an Anti-nerve Growth Factor Antibody (Frunevetmab) for the Treatment of Degenerative Joint Disease-Associated Chronic Pain in Cats: A Multisite Pilot Field Study
Source: Front Vet Sci. 2021 May 28;8:610028. doi: 10.3389/fvets.2021.610028 (PMC8195238; doi:10.3389/fvets.2021.610028)

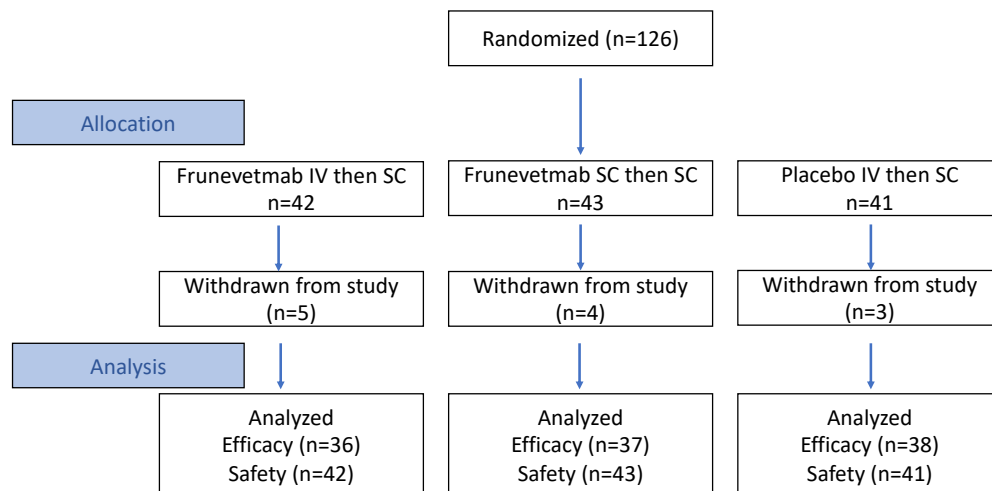

Supplementary Figure 1. Diagram showing flow of cases through the study.

Supplement: Supplementary file 1 [file Data_Sheet_1.PDF]
