# Supplementary material for: Efficacy and Safety of an Anti-nerve Growth Factor Antibody (Frunevetmab) for the Treatment of Degenerative Joint Disease-Associated Chronic Pain in Cats: A Multisite Pilot Field Study
Source: Front Vet Sci. 2021 May 28;8:610028. doi: 10.3389/fvets.2021.610028 (PMC8195238; doi:10.3389/fvets.2021.610028)
Supplement: Supplementary file 2 [file Table_1.docx]

# Supplementary Table 1a: Analysis population for safety and CSOM assessments

|  | | **Number of Cats** | | |
| --- | --- | --- | --- | --- |
|  |  | **Placebo** | **Frunevetmab** | |
| Safety population | | 41 | 85 | |
|  | | Group 1 | Group 2 (IV/SC) | Group 3 (SC/SC) |
| CSOM  Success/Failure | Day 14 | 35  (85.4%) | 36  (85.7%) | 37  (86.0%) |
|  | Day 28 | 38  (92.7%) | 36  (85.7%) | 382  (88.4%) |
|  | Day 42 | 38  (92.7%) | 34  (81.0%) | 372  (86.0%) |
|  | Day 56 | 381  (92.7%) | 33  (78.6%) | 382  (88.4%) |
| CSOM Continuous Variable | Day 14 | 35  (85.4%) | 36  (85.7%) | 37  (86.0%) |
|  | Day 28 | 38  (92.7%) | 36  (85.7%) | 37  (86.0%) |
|  | Day 42 | 38  (92.7%) | 34  (81.0%) | 36  (83.7%) |
|  | Day 56 | 37  (90.2%) | 33  (78.6%) | 37  (86.0%) |
| CSOM Global Assessment | Day 28 | 38  (92.7%) | 36  (85.7%) | 38  (88.4%) |
|  | Day 56 | 37  (90.2%) | 32  (76.2%) | 38  (88.4%) |

1 Includes 1 cat withdrawn at Day 42 due to perceived lack of efficacy and categorized as a treatment failure at Day 56

2 Includes 1 cat withdrawn at Day 14 due to perceived lack of efficacy and categorized as a treatment failure at Days 28, 42 and 56

# Supplementary Table 1b: Analysis population for FMPI and orthopedic examination

|  | | **Number of Cats** | | |
| --- | --- | --- | --- | --- |
|  |  | **Placebo** | **Frunevetmab** | |
|  | | Group 1 | Group 2 | Group 3 |
| FMPI  Success/Failure | Day 14 | 37  (90.2%) | 37  (88.1%) | 36  (83.7%) |
|  | Day 28 | 38  (92.7%) | 36  (85.7%) | 382  (88.4%) |
|  | Day 42 | 37  (90.2%) | 34  (81.0%) | 352  (81.4%) |
|  | Day 56 | 371  (90.2%) | 33  (78.6%) | 372  (86.0%) |
| FMPI Percent Total Score | Day 14 | 37  (90.2%) | 37  (88.1%) | 36  (83.7%) |
|  | Day 28 | 38  (92.7%) | 36  (85.7%) | 37  (86.0%) |
|  | Day 42 | 37  (90.2%) | 34  (81.0%) | 34  (79.1%) |
|  | Day 56 | 36  (87.8%) | 33  (78.6%) | 36  (83.7%) |
| FMPI Questions 18, 19, 20, 21 | Day 14 | 37  (90.2%) | 37  (88.1%) | 36  (83.7%) |
|  | Day 28 | 38  (92.7%) | 36  (85.7%) | 37  (86.0%) |
|  | Day 42 | 37  (90.2%) | 34  (81.0%) | 34  (79.1%) |
|  | Day 56 | 36  (87.8%) | 33  (78.6%) | 36  (83.7%) |
| Orthopedic Examination | Day 28 | 38  (92.7%) | 36  (85.7%) | 37  (86.0%) |
|  | Day 56 | 36  (87.8%) | 33  (78.6%) | 36  (83.7%) |

1 Includes 1 cat withdrawn at Day 42 due to perceived lack of efficacy and categorized as a treatment failure at Day 56

2 Includes 1 cat withdrawn at Day 14 due to perceived lack of efficacy and categorized as a treatment failure at Days 28, 42 and 56
