# Supplementary material for: Efficacy and Safety of an Anti-nerve Growth Factor Antibody (Frunevetmab) for the Treatment of Degenerative Joint Disease-Associated Chronic Pain in Cats: A Multisite Pilot Field Study
Source: Front Vet Sci. 2021 May 28;8:610028. doi: 10.3389/fvets.2021.610028 (PMC8195238; doi:10.3389/fvets.2021.610028)
Supplement: Supplementary file 3 [file Table_2.docx]

# Supplementary Table 2: Details of subjects withdrawn from the study due to safety concerns

| **Treatment** | **Case ID** | **Reason** | **Comments** |
| --- | --- | --- | --- |
| Frunevetmab  Day 0:IV, Day 28:SC | 01-13 | Adverse Event [localized skin reaction associated with collar] | Dermatitis around neck |
| Frunevetmab  Day 0:IV, Day 28:SC | 03-15 | Adverse Event [localized skin reaction associated with collar] | Sore on neck from collar |
| Frunevetmab Day 0:IV, Day 28:SC | 04-04 | Adverse Event [decreased activity, decreased appetite,  anaemia, thrombocytopenia, death] | Diagnosed with immune- mediated hemolytic  anemia and thrombocytopenia |
| Placebo | 11-15 | Other | Sudden death |
| Frunevetmab Day 0:IV, Day 28:SC | 11-01 | Owner withdrew consent | Sore on neck from collar |
| Frunevetmab Day 0:IV, Day 28:SC | 14-11 | Owner withdrew consent | Development of aural mass |
