# Supplementary material for: Efficacy and Safety of an Anti-nerve Growth Factor Antibody (Frunevetmab) for the Treatment of Degenerative Joint Disease-Associated Chronic Pain in Cats: A Multisite Pilot Field Study
Source: Front Vet Sci. 2021 May 28;8:610028. doi: 10.3389/fvets.2021.610028 (PMC8195238; doi:10.3389/fvets.2021.610028)
Supplement: Supplementary file 4 [file Table_3.docx]

**Supplementary Table 3:** Selected serum chemistry (hepatic, renal) and urinalysis results at screening and study exit (Day 56)

|  | **Frunevetmab** | | | | | | **Placebo** | | |  |  |
| --- | --- | --- | --- | --- | --- | --- | --- | --- | --- | --- | --- |
|  | **Day 0:IV Day 28:SC** | | | **Day 0:SC Day 28:SC** | | |  |  |  | **P-value*** | **Normal**  **Reference Range** |
| **VARIABLE** | **N** | **Mean** | **SD** | **N** | **Mean** | **SD** | **N** | **Mean** | **SD** |  |  |
| ALT (SGPT) SCREEN | 42 | 50.19 | 38.17 | 43 | 57.72 | 30.06 | 41 | 49.95 | 30.19 | 0.1066 (r) | 27-158 U/L |
| ALT (SGPT) DAY 56 | 36 | 52.72 | 35.06 | 39 | 60.79 | 45.44 | 37 | 51.73 | 35.32 |  |  |
| AST (SGOT) SCREEN | 42 | 25.60 | 15.02 | 43 | 27.56 | 10.86 | 40 | 24.60 | 8.19 | 0.3160 (r) | 16-67 U/L |
| AST (SGOT) DAY 56 | 36 | 25.53 | 10.40 | 39 | 29.08 | 14.31 | 37 | 23.22 | 8.26 |  |  |
| Alk  Phosphatase SCREEN | 42 | 22.55 | 12.55 | 43 | 24.67 | 12.57 | 41 | 26.95 | 18.61 | 0.2089 (r) | 12-59 U/L |
| Alk Phosphatase DAY 56 | 36 | 22.72 | 12.58 | 39 | 24.85 | 15.78 | 37 | 28.11 | 19.64 |  |  |
| Total Bilirubin SCREEN | 42 | 0.11 | 0.06 | 43 | 0.11 | 0.04 | 41 | 0.10 | 0.03 | 0.9403 (r) | 0-0.3 mg/dL |
| Total Bilirubin DAY 56 | 36 | 0.12 | 0.04 | 39 | 0.13 | 0.05 | 37 | 0.12 | 0.04 |  |  |
| Urea Nitrogen SCREEN | 42 | 28.45 | 6.36 | 43 | 29.12 | 9.39 | 41 | 31.34 | 9.93 | 0.5720 (r) | 16 – 37 mg/dL |
| Urea Nitrogen DAY 56 | 36 | 29.94 | 7.03 | 39 | 31.21 | 8.79 | 37 | 31.95 | 9.80 |  |  |
| Creatinine  SCREEN | 42 | 1.53 | 0.40 | 43 | 1.68 | 0.47 | 41 | 1.70 | 0.51 | 0.1979 (r) | 0.9 - 2.5 mg/dL |
| Creatinine DAY 56 | 36 | 1.49 | 0.38 | 39 | 1.72 | 0.53 | 37 | 1.65 | 0.51 |  |  |
| BUN:Creatinine Ratio SCREEN | 42 | 19.19 | 4.11 | 43 | 17.71 | 4.95 | 41 | 18.71 | 4.00 | 0.1316 (r) | Ratio |
| BUN:Creatinine Ratio DAY 56 | 36 | 20.70 | 4.54 | 39 | 18.89 | 5.42 | 37 | 19.76 | 3.73 |  |  |
| Total Protein SCREEN | 42 | 7.23 | 0.57 | 43 | 7.19 | 0.60 | 41 | 7.14 | 0.58 | 0.7641 | 6.3 – 8.8 g/dL |
| Total Protein DAY 56 | 36 | 7.34 | 0.53 | 39 | 7.35 | 0.64 | 37 | 7.33 | 0.70 |  |  |
| Albumin SCREEN | 42 | 3.09 | 0.29 | 43 | 3.05 | 0.32 | 41 | 3.06 | 0.33 | 0.9705 (r) | 2.6 – 3.9 g/dL |
| Albumin DAY 56 | 36 | 3.17 | 0.28 | 39 | 3.06 | 0.32 | 37 | 3.19 | 0.24 |  |  |
| SDMA SCREEN | 42 | 10.74 | 4.20 | 43 | 11.86 | 4.03 | 41 | 11.17 | 3.92 | 0.2671 (r) | 0.0 – 14.0 ug/dL |
| SDMA DAY 56 | 36 | 11.17 | 4.25 | 39 | 13.31 | 5.74 | 37 | 12.11 | 3.84 |  |  |
| Urine Specific Gravity SCREEN | 40 | 1.036 | 0.01 | 38 | 1.032 | 0.02 | 37 | 1.033 | 0.02 | 0.4049 (r) | Not provided |
| Urine Specific Gravity DAY 56 | 29 | 1.037 | 0.01 | 36 | 1.037 | 0.01 | 31 | 1.034 | 0.01 |  |  |

*P-values generated for the changes from Screening by repeated measures analysis of covariance with Treatment, Study Day and Treatment by Study Day as Fixed effects, Day 0 as covariate with Site and Treatment by Site as Random effects.

Note that p-values are presented only for parameters with statistically significant Treatment by Study Day or Treatment effects. (r) indicates values were ranked prior to ANCOVA.
